# Use of platelet-rich plasma (PRP) and injectable platelet-rich fibrin (i-PRF) in oral lichen planus treatment: a systematic review of randomized controlled trials

**DOI:** 10.1186/s12903-025-06189-7

**Published:** 2025-05-28

**Authors:** Wojciech Niemczyk, Katarzyna Janik, Stanisław Niemczyk, Jacek Żurek, Edward Lynch, Steven Parker, Mark Cronshaw, Dariusz Skaba, Rafał Wiench

**Affiliations:** 1 Department of Periodontal Diseases and Oral Mucosa Diseases, Faculty of Medical Sciences in Zabrze, Medical University of Silesia, Pl. Traugutta 2, Zabrze, 41-800 Poland; 2 Municipal Hospital No. 4 in Gliwice, Zygmunta Starego 20, Gliwice, 44-100 Poland; 3 Specialist Medical Practice “Clinical Periodontology”, Żuradzka 15 Street, Olkusz, 32-300 Poland; 4 School of Pharmacy, De Montfort University, Leicester, LE1 9BH UK

**Keywords:** Blood platelets, Lichen planus, Oral lichen planus, Periodontics, Platelet-rich fibrin, Platelet-rich plasma

## Abstract

**Background:**

Lichen planus (LP) is a chronic inflammatory disease that affects the skin and mucous membranes, including the oral cavity. The prevalence of oral lichen planus (OLP) in the general population is estimated to be between 0.5% and 2%, with a higher incidence observed in women aged between 30 and 80 years. The etiology of OLP remains unclear, which presents a significant challenge in terms of diagnosis and treatment. This systematic review assessed the efficacy of platelet-rich plasma (PRP) and injectable platelet-rich fibrin (i-PRF) in the treatment of OLP.

**Methods:**

A comprehensive literature search was conducted via multiple databases in accordance with the PRISMA 2020 guidelines.

**Results:**

A total of seven randomized controlled trials were subjected to analysis. It has been demonstrated that both PRP and i-PRF have the capacity to significantly improve clinical outcomes, including pain and lesion severity. However, the majority of studies have not demonstrated statistically significant differences between PRP/i-PRF and corticosteroid treatments.

**Conclusions:**

While PRP and i-PRF demonstrate potential in alleviating symptoms and promoting tissue healing, their effectiveness appears to be analogous to that of corticosteroids in the majority of RCTs. Further high-quality, standardized studies are required to substantiate their function as alternative or adjunctive therapies in the management of OLP.

**Trial registration:**

This systematic review was registered in the International Prospective Register of Systematic Reviews (PROSPERO) on 28 November 2024 (PROSPERO 2024 CRD42024615291).

## Background

Lichen planus (LP) is a chronic or recurrent inflammatory disease affecting the skin and some of its appendages, as well as the mucosa, including the oral cavity [1]. The prevalence of oral lichen planus (OLP) is 0.5–2% in the general population, with a higher incidence among women aged 30–80 years. The etiology remains uncertain, which presents challenges in the diagnosis and treatment of this condition. Several potential risk factors and triggers have been identified, including local and systemic inducers of cell-mediated hypersensitivity, infection, the autoimmune response to epithelial antigens, and stress or trauma [2, 3]. Further investigation of the aforementioned factors may facilitate improvements in the diagnostic process, particularly in cases where malignancy is a concern. Salivary markers may include exosomal microRNAs, complement fraction C3c, fibrinogen fragment D, cystatin SA, p53, MCL-1, BMI1, p16, matrix metalloproteinases (MMPs), tumor necrosis factor α (TNF-α), and interleukin 6 (IL-6), among others. Furthermore, cyclooxygenase-2 (COX-2) [4–7] and tenascin-C [8], which are long noncoding RNAs and genes associated with altered autophagy are additional factors that should be considered [9–11]. The clinical presentation allows for the classification of the condition into three subtypes (reticular, atrophic, and ulcerative or erosive) [3, 12] or six subtypes (reticular, papular, plaque, atrophic, bullous, erosive) [13]. The erosive form has been demonstrated to be most prevalent among smokers [14, 15]. Most lesions manifest in the buccal region; however, other areas of the oral mucosa may also be involved. The most prevalent subtype is the reticular subtype, which is typically asymptomatic and presents with distinctive Wickham striae. Conversely, atrophic and erosive subtypes may result in discomfort and symptoms such as oral burning, soreness, or pain, which may manifest spontaneously or during activities such as chewing or toothbrushing [16]. Evidence has demonstrated that long-term gingival OLP can result in gingival recession, which necessitates periodontal surgery [17, 18]. Furthermore, such lesions are susceptible to malignant transformation, with an estimated risk of 1% to 2% [3]. Given the uncertain etiology of OLP, a range of therapeutic approaches may be considered, depending on the severity of the disease. These include the use of topical anesthetics, such as benzdamine (liquid/spray), as well as steroids administered topically, orally, via intralesional injections, or systemically. The aforementioned therapeutic approaches include the use of ointments, creams, adherent pastes, immunosuppressants or immunomodulatory agents, psoralen plus ultraviolet-A radiation (PUVA) therapy, photodynamic therapy (PDT), laser therapy (photobiomodulation), hyaluronic acid application, and finally, the use of platelet concentrates [2, 16, 19–27]. Maintaining an optimal level of oral hygiene and preventing any mucosal trauma at each stage of the treatment process are highly important, as these measures may contribute to the control of lesion severity [19, 22]. Platelet-rich plasma (PRP) is an autologous blood product obtained by sequestering and concentrating platelets through gradient-density centrifugation [28, 29]. The resulting concentrate contains a concentration of platelets that exceeds normal physiological levels [30]. The role of platelets in the initial stages of wound regeneration is pivotal, as they facilitate primary coagulation [31] and subsequently stimulate the natural healing cascade [32, 33]. They facilitate tissue repair through the release of active biomolecules, including platelet-derived growth factor (PDGF), transforming growth factors (TGF-β1 and TGF-β2), insulin-like growth factor (IGF) and epidermal growth factor (EGF) [28, 31]. Furthermore, platelets contain and, upon activation, secrete proteins, including fibrin, fibronectin, and vitronectin, which are instrumental in a significant portion of the cell adhesion process, epithelial migration, and the matrix for bone and connective tissue [28]. Consequently, they are instrumental in cell proliferation, collagen production and osteoid formation [34]. The advancement of manufacturing techniques has resulted in the identification of injectable platelet-rich fibrin (i-PRF), which is currently available in a liquid form. This is due to the production process, which does not require the addition of anticoagulants or other additives [35]. One of the most significant advantages of i-PRF is its capacity to secrete growth factors, including PDGF, TGF-β and IGF-I [36, 37], which increase cell migration by inducing the expression of proteins such as type I collagen and transforming growth factor mRNA [38]. Compared with the application of PRP, the implementation of i-PRF has provided the opportunity for superior clinical outcomes in many areas of medicine [39, 40]. In the field of dentistry, a substantial body of research has been conducted on the utilization of APC applications across various specialties. The most prevalent application of platelet concentrates is in the field of surgery, where their efficacy in accelerating post-extraction healing and mitigating discomfort in various surgical procedures has been well-documented. The utilization of APCs has been demonstrated in the domain of orthodontics, facilitating expedited movement of teeth. In the field of endodontics, APCs have been employed to enable revascularization or the withdrawal of pulp inflammation [40]. The authors of the study identified a dearth of systematic reviews focusing on the efficacy of PRP and i-PRF in lichen planus therapy on the basis of randomized controlled trials (RCTs) with appropriate assessment of the risk of bias. The extant literature on this topic exhibits a paucity of consistency in the results obtained, prompting the authors to undertake a comprehensive review with a view to emphasizing thorough research outcomes. The objective of this study was to ascertain whether PRP and i-PRF can mitigate the severity of the disease when used as adjunctive treatments for LP.

## Materials and methods

In accordance with the PRISMA guidelines, the protocol for this systematic review was registered in the International Prospective Register of Systematic Reviews (PROSPERO) on 28 November 2024 (PROSPERO 2024 CRD42024615291).

### Focused questions

A systematic review was conducted in accordance with the PICO framework [41]. In patients with oral lichen planus (Population), is platelet-rich plasma or injectable platelet-rich fibrin (Intervention) more effective than classic known pharmacological treatment methods (Comparison) in reducing pain, burning symptoms, and clinical presentation (Outcome)?

### Search strategy

The review was conducted following the Preferred Reporting Items for Systematic Reviews and Meta-Analyses (PRISMA 2020) guidelines [42]. The electronic literature search was conducted via the MEDLINE (PubMed), Embase, Google Scholar, and Scopus databases from inception until 3 November 2024. The keywords and Boolean operators used in the searches of Scopus and Google Scholar were (“prp” OR “platelet AND rich AND plasma” OR “injectable AND platelet AND rich AND fibrin” OR “i-prf”) AND (“lichen AND planus”) AND (“treatment”). The terms used in the Embase database were (‘prp’/exp OR ‘prp’ OR ‘platelet rich plasma’/exp OR ‘platelet rich plasma’ OR ‘injectable platelet-rich fibrin’ OR ‘i-prf’) AND (‘lichen planus’/exp OR ‘lichen planus’) AND (‘treatment’/exp OR ‘treatment’). The terms from the PubMed database search were (“PRP” OR “Platelet-rich plasma” OR “injectable platelet-rich fibrin” OR “i-PRF”) AND (“Lichen planus” OR “Oral lichen planus”) AND (“Treatment”). Furthermore, the authors conducted a “snowball” search to identify additional studies by examining the bibliographies of publications selected for full-text review. Furthermore, Google Scholar was employed to corroborate the veracity of the cited studies. The electronic search was limited to studies published in the English language to comply with the inclusion criteria. To minimize the potential for bias in the search process, the authors elected not to restrict the search to randomized controlled trials. They recognized that the classification of academic papers is not always accurate, particularly for recent publications that may not yet be appropriately categorized. The databases were searched by three authors (W.N., R.W., and K.J.) via identical sets of search terms. Once the potential studies had been identified, all the authors collectively reviewed them to ascertain their suitability for inclusion. To collate the data from the included studies, two authors (W.N. and K.J.) conducted a collaborative literature search to gather the necessary data.

### Selection of studies

The objective of this systematic review was to assess the potential use of PRP and its derivative, i-PRF, in the treatment of lichen planus. The hypothesis was that autologous platelet concentrates in the form of PRP and i-PRF could serve as an alternative to the currently used topical medications for oral lichen planus. The criteria for the inclusion of articles and the exclusion of articles from this review are presented in Table 1.

**Table 1:** Selection criteria for papers included in the systematic review

Inclusion criteria	Exclusion criteria
Randomized controlled trials	Case reports/Case series
Full text available	Narrative reviews
Human studies	Systematic reviews
English language	Meta-analysis
Patients aged ≥18 years	Non-English language publications
Nonsmoking patients	High risk of bias of the study
Low or moderate risk of bias	Letters to Editor
	Animal studies
	Studies on smoking patients
	Conference papers

### Data extraction

#### Risk of bias in individual studies

In the initial phase of the review selection process, each reviewer was responsible for independently assessing titles and abstracts. This approach was adopted to minimize the potential influence of bias in the assessment procedure. To ascertain the level of agreement among the reviewers, the authors employed a tool known as Cohen’s k test [43]. In the event of a disagreement regarding the inclusion or exclusion of a study within the scope of the review, the relevant authors engaged in discussion until a consensus was reached.

#### Quality assessment

Two independent reviewers, W.N. and R.W., were responsible for conducting quality assessments of the included studies. The evaluation of study design, implementation, and analysis was conducted in accordance with the following criteria: whether patients were randomized to their groups, whether the study was split-mouth, whether the number of patients studied was based on a precalculated necessary number of patients, whether the study groups were balanced to within 10% variation between groups, whether the method of obtaining PRP or i-PRF was accurately described, whether the authors described in detail the method of administration, including the duration and frequency of administration, whether the diagnosis of lichen planus was made on the basis of histopathological examination, and whether tests of blood parameters with particular reference to platelet counts were performed prior to patient inclusion in the study. In accordance with the criteria established for this evaluation, a total score below 3 was deemed indicative of a high probability of bias. A score of 4–6 points indicated a moderate risk of bias, whereas a score of 7 points or above indicated a low risk of bias. Any discrepancies in scoring were resolved through discussion until a consensus was reached.

#### Risk of bias across studies assessment

The scores for each study were calculated, and the overall risk of bias (low, moderate, or high) for each study was determined. This process was carried out in accordance with the recommendations set forth by the Cochrane Handbook for Systematic Reviews of Interventions [44].

#### General characteristics of the studies assessment

From each article, the authors extracted the following information (apart from that used to determine the risk of bias): the country in which the study was conducted, the number of patients, their sex, the age range and its mean with standard deviation, the treatment in each group in the study, the frequency and duration of administration, the indicators used to assess the results of the study, the main results and the follow-up period.

## Results

### Study selection

**Fig. 1:**
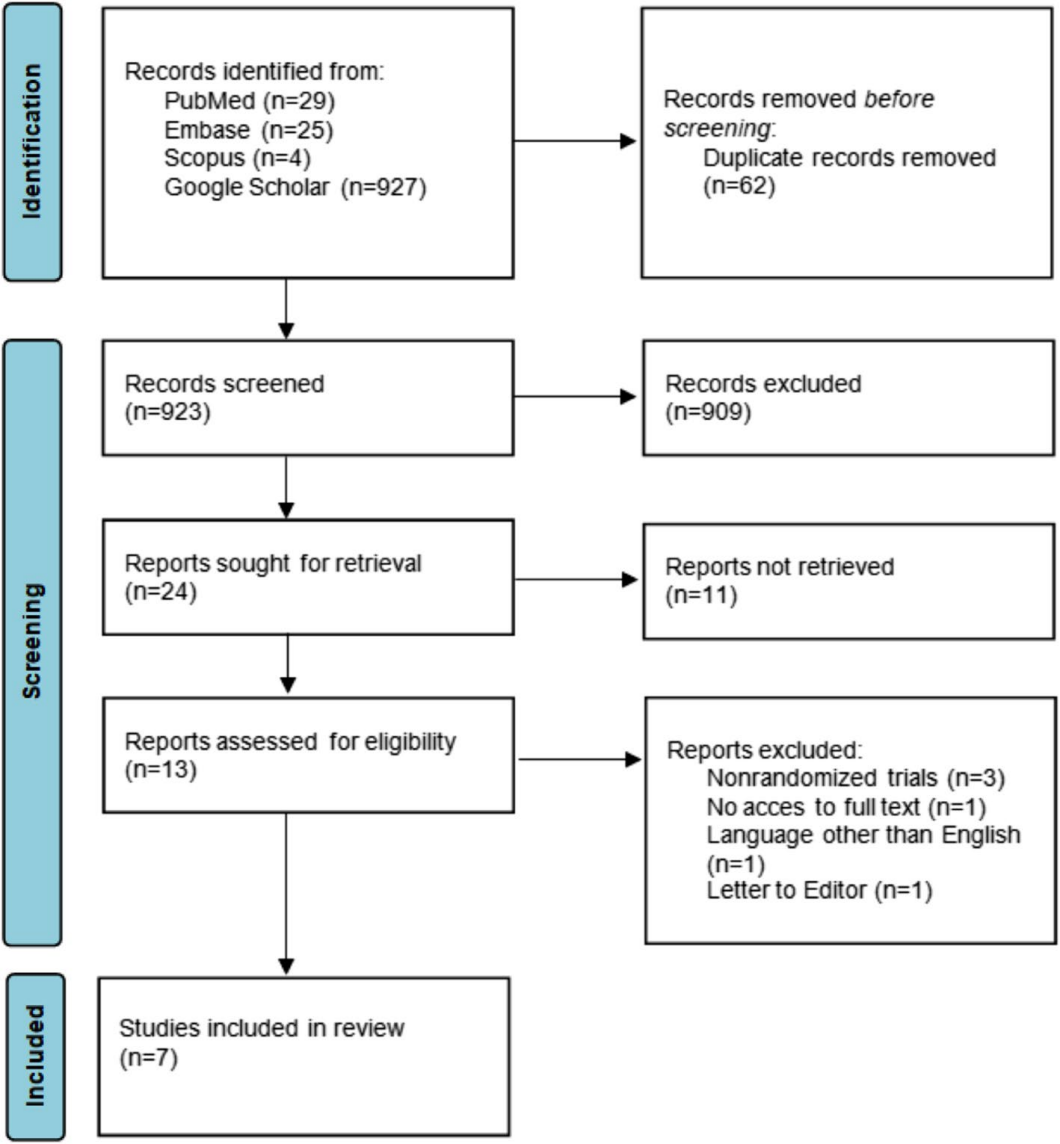
PRISMA 2020 flow diagram

In the first stage of the search for articles to be included that met the criteria, duplicates were removed, and 923 articles remained. After an initial review of the title and abstract, 24 articles were eligible for full-text review. Thirteen reports were assessed for eligibility. On the basis of the inclusion and exclusion criteria in Table 1, 6 articles were rejected, and 7 were included in the analyses. A flowchart of the research approach according to the PRISMA 2020 statement [42] is shown in Fig. 1.

Among the seven included trials, the oldest date was 2021. Among the trials, 2 each were conducted in India and Egypt, and 1 each was conducted in Italy, Syria, and Turkey. Four of the 7 studies were conducted in a split-mouth format. The details are shown in Table 2.

**Table 2:** A general overview of the studies

Author and Year	Country	Study Design	Split-Mouth
Sharma et al. (2023) [45]	India	Randomized Controlled Clinical Trial	Yes
Sethi Ahuja et al. (2020) [46]	India	Randomized Controlled Clinical Trial	No
Shalby et al. (2022) [47]	Egypt	Randomized Controlled Clinical Trial	No
Saglam et al. (2021) [48]	Turkey	Randomized Controlled Clinical Trial	Yes
Al‐Hallak et al. (2023) [49]	Syria	Randomized Controlled Clinical Trial	Yes
Hijazi et al. (2022) [50]	Egypt	Randomized Controlled Clinical Trial	No
Bennardo et al. (2021) [51]	Italy	Randomized Controlled Clinical Trial	Yes

### Risk of bias across studies

The analysis included seven articles. Six studies had a low risk of bias, and one study had a moderate risk of bias. No single study achieved a maximum score of 9. None of the studies were excluded because of the high risk of bias. The most common criterion not met was the completion of blood tests before treatment. Only three studies met these criteria. A single point was awarded for a positive response. Conversely, no additional points were assigned in instances of a negative or uncertain response. Table 3 presents the exact level of bias in each included study.

**Table 3:** The results of the quality assessment and risk of bias across the studies

Study							
Criteria	Sharma et al. (2023) [45]	Sethi Ahuja et al. (2020) [46]	Shalby et al. (2022) [47]	Saglam et al. (2021) [48]	Al‐Hallak et al. (2023) [49]	Hijazi et al. (2022) [50]	Bennardo et al. (2021) [51]
Random allocation							
Split-mouth study type							
Calculated study group							
Balanced study groups (+/- 10%)							
Clear method of obtaining i-PRF/PRP							
Well-defined method of i-PRF administration with number of sessions							
Inclusion/exclusion criteria clearly defined							
Both clinical and histopathological diagnosis							
Blood tests of patients before examination with platelet count assessment							
Total	5	7	8	8	7	7	8
Risk of bias	Moderate	Low	Low	Low	Low	Low	Low

*i-PRF* injectable platelet-rich fibrin, *PRP* platelet-rich plasma

### General characteristics of the included studies

In four of the seven trials, the number of patients needed was calculated before the trial. The number of patients ranged from 9--29, and in each of the included studies, the number of female patients exceeded the number of male patients. Detailed data on the number of patients, their sex, and their age are shown in Table 4.

**Table 4:** Characteristics of the patients in the study

Author/Year	Sample Size Calculation	Patients	Lichen Planus Type	Sex		Age (Years)	
				Female	Male	Mean (±SD)	Range
Sharma et al. (2023) [45]	No	20	Only erosive	15	5	35.75 ± 8.75	18–60
Sethi Ahuja et al. (2020) [46]	No	20	Only erosive	18	2	44.5	28–60
Shalby et al. (2022) [47]	Yes	29	Only erosive	18	11	43.2 ± 10.1	35–54
Saglam et al. (2021) [48]	Yes	24	Only erosive	14	10	52.25	34–76
Al‐Hallak et al. (2023) [49]	No	12	24 lesions in 12 patients: 2-erosive 1-ulcerative 9-reticular/plaque 8-ulcerative white striae 4-white striae with atrophic area	9	3	48 ± 12.7	No data
Hijazi et al. (2022) [50]	Yes	20	Only erosive	18	2	42.60 ± 11.46	24–65
Bennardo et al. (2021) [51]	Yes	9	7 patients had white striae with bilateral atrophic areas 2 patients had pure reticular clinical pattern	6	3	59.56 ± 3.57	55–64

*SD * Standard deviation

### Study outcomes

Among the included trials, 3 compared PRP with triamcinolone acetonide [41, 42, 46], 2 compared i-PRF with triamcinolone acetonide [45, 47], and 1 compared i-PRF with methylprednisolone acetate [44]. All study groups in the above trials received injectable formulations. Only the study by Shalby et al investigated a platelet lysate mucoadhesive gel based on PRP, which was in gel form, as was the control group in the same study, which received a placebo. The gel was applied three times a day for a period of four weeks [43], while PRP and i-PRF injections were administered every week for a period of four weeks [41, 45–47], eight weeks [42], or four times every 14 days [44]. All the studies analyzed employed the use of the visual analog scale (VAS) to assess pain [41–47], with some additionally utilizing the VAS to assess the burning sensation [41, 42]. Three studies employed the Thongprasom sign score to assess the clinical picture [43, 46, 47]. Al-Hallak employed the RUE (reticulation/keratosis, ulceration, erythema) score, which encompasses reticulation/keratosis, erythema, and ulceration, to assess the clinical presentation [45]. In all studies, both PRP and i-PRF significantly reduced pain, as well as indices indicating size, erythema, or ulceration. In contrast, all studies demonstrated no statistically significant difference between the study groups. The exception was the study by Shalby et al., which consisted of a test group and a placebo control group. This is the sole study in which the platelet concentrate-based preparation demonstrated superiority over the study group [43]. The detailed data for each of the included studies are shown in Table 5.

**Table 5:** Detailed characteristics of the studies included in this review

Author/Year	Treatment Groups	Number of i-PRF/PRP Administration Sessions	Evaluation	Main Results	Changes in VAS of pain	Follow-up Period
Sharma et al. (2023) [45]	1.Triamcinolone acetonide injection 2. PRP	Injections weekly for month	- Pain and burning sensation with VAS - Size of the lesion - Severity of the lesion with distinction: (Completely resolved, Keratosis only, Mild erythema, Moderate erythema, Ulceration)	In OLP, triamcinolone acetonide and PRP are equally effective in reducing lesion size. However, PRP is demonstrably more effective than intralesional triamcinolone acetonide in reducing the severity of the lesion and alleviating pain and burning sensations in patients with OLP.	1. 7.45±1.19 to 0.10±0.31 2. 7.45±1.19 to 0.70±0.87	4 months
Sethi Ahuja et al. (2020) [46]	1.Triamcinolone acetonide injection 2. PRP	Injections weekly for 8 weeks	- Pain and burning sensation with VAS - Size of the lesion - Severity of the lesion with distinction: (mild erythema, moderate erythema, severe erythema, ulceration)	There were notable decreases in pain scores, lesion size, and erythema in both the steroid and PRP groups over the four-month observation period. The reduction in pain was 82.55% in the steroid group and 93.5% in the PRP group. Similarly, the reduction in erythema was 91.66% in the steroid group and 93.33% in the PRP group at the two-month mark. Notwithstanding these trends, the comparative p values between the groups were found to be insignificant.	1. 8.70±0.94 to 1.60±2.27 2. 8.90±0.99 to 0.60±0.96	4 months
Shalby et al. (2022) [47]	1. Platelet lysate mucoadhesive gel (Based on PRP) 2. Placebo gel	Using gel 3 times per day for 4 weeks	- Pain with VAS - Clinical picture according to Thongprasom sign score	The patients in both groups showed a statistically significant reduction in all the assessed parameters of erosive lichen planus from baseline to 4 months of treatment and follow-up. However, when we compared the pain reduction, lesion size and erythema scores between the two groups, we found that the difference was not statistically significant.	No exact data available	3 months
Saglam et al. (2021) [48]	1.Methylprednisolone acetate injection 2. i -PRF	4 sessions at 15-day intervals	- VAS for pain and satisfaction - OHIP-14 - Objective evaluation of lesion size	The intragroup comparisons revealed a statistically significant reduction in VAS-pain and lesion size in both the i-PRF group (from 81.88±17.74 to 13.33±18.34, and from 4.79±0.41 to 1.88±1.08, respectively) and the corticosteroid group (from 80.21±17.35 to 23.33±26.81, and from 4.71±0.46 to 2.21±1.35, respectively) in the sixth month compared to the baseline (*p*<0.001). Furthermore, there was a notable increase in VAS satisfaction in both the i-PRF group (from 26.67±17.8 to 85.63±16.24) and the corticosteroid group (from 28.33±17.05 to 74.38±24.11) in the sixth month compared to baseline (*p*<0.001). Nevertheless, no significant intergroup differences were observed in any of the values.	1. 80.21±17.35 to 23.33±26.81 2. 81.88±17.74 to 13.33±18.34	6 months
Al‐Hallak et al. (2023) [49]	1.Triamcinolone acetonide injection 2. i-PRF	Injections weekly for 4 weeks	- VAS for pain REU score Lesion areas	Both injectable TA and i-PRF were found to be effective in the management of oral lichen planus. Following a four-week course of treatment, there was an average reduction in the VAS score (68.5% i-PRF, 91% TA) and an average reduction in the REU score (74% i-PRF, 91% TA). There were no statistically significant differences between the two treatment methods (*p* > 0.05).	1. 6.33±2.26 to 0.58±0.79 2. 6.08±2.15 to 1.91±1.31	3 months
Hijazi et al. (2022) [50]	1.Triamcinolone acetonide injection 2. PRP	Injections weekly for 4 weeks	- Pain with VAS - Clinical picture according to Thongprasom sign score	Both groups demonstrated a significant improvement in clinical parameters, including pain and clinical score, with a *p* value of 0.001. Regarding the remission of lesions, 80% of patients in the PRP group demonstrated complete remission, in comparison to 70% in the TA group. Nevertheless, no statistically significant differences were observed between the two groups with regard to pain score, clinical score, or remission.	No exact data available	3 months
Bennardo et al. (2021) [51]	1.Triamcinolone acetonide injection 2. i-PRF	Injections weekly for a month	- Pain with VAS - Clinical picture according to Thongprasom sign score	Four weeks following the final injections, an average reduction of 59.8% in lesion extension and an average reduction of 47.6% in VAS score for PRF-treated sites was observed. The corresponding figures for TA-treated sites were 59.2% and 40%, respectively. There were no statistically significant differences between the two groups.	1. 4.6±2.5 to 1.9±1.5 2. 5.9±2.0 to 2.9±2.1	3 months

*PRP* platelet-rich plasma, *VAS* visual analog scale, *OLP* oral lichen planus, *TA* triamcinolone acetonide, *i-PRF* injectable platelet-rich fibrin, *OHIP-14* oral health impact profile-14, *RUE* Reticulation/keratosis, erythema, and ulceration

## Discussion

This systematic review explored the use of PRP and injectable PRF in the treatment of oral lichen planus. The etiology of oral lichen planus is multifactorial and has not yet been fully elucidated. However, it may be associated with immune, genetic, endocrine, psychosomatic or microcirculation disorders, along with trace element deficiency [52, 53]. Furthermore, viral infections, including hepatitis C, Epstein–Barr, and varicella-zoster herpes viruses, as well as human papillomaviruses (types 16 and 18), have been shown to exacerbate the severity of pathological symptoms [53–55]. An increasing body of evidence emphasizes the vital role of immune dysregulation and the extensive release of multiple inflammatory mediators and cells in the pathogenesis of OLP. One potential mechanism is that T lymphocytes may be activated by the presentation of antigens through major histocompatibility complex (MHC) molecules, which can induce keratinocyte apoptosis. An alternative, nonspecific hypothesis posits that the cause lies in the expression of matrix metalloproteinases and mast cell degranulation. These phenomena result in the progressive accumulation of T cells, the destruction of the basement membrane, and the apoptosis of keratinocytes. In the progressive phase, a high level of antigen-presenting cells and T lymphocytes is observed, which amplifies the induction of an inflammatory response. This is in contrast to advanced stages, where the predominance is CD8+ T cells [52, 53]. The various interactions between macrophages, dendritic cells, mast cells, CD8+ T cells, Th cells, keratinocytes, and fibroblasts perpetuate and reinforce the chronic characteristics of the disease. The chronicity of OLPs, with periods of exacerbation and remission, necessitates prolonged administration of medications [19, 26]. The findings of the seven studies included in this systematic review demonstrate a notable reduction in pain, lesion size, erythema, and ulcerations when products containing PRP or i-PRF are employed in the treatment of oral lichen planus. Conversely, no statistically significant differences were observed between the study groups in the majority of the studies. The trial conducted by Shalby et al. did not yield statistically significant differences between the study groups. However, notably, this experiment involved only the study group receiving the PRP-based product and the control group receiving a placebo [47]. Notably, the use of autologous platelet concentrates is associated with a reduced risk of complications compared with the use of corticosteroids. The most common example is candidiasis, followed by oral mucosa thinning. Additionally, discomfort may occur during local therapy. In cases of severe multifocal atrophic or ulcerative lesions, the systemic administration of corticosteroids is indicated. However, this can lead to major complications, including adrenal suppression, hypertension, hyperglycemia, weight gain, gastrointestinal irritation, mood alterations, insomnia, and osteoporosis. The risk of these adverse effects increases with systemic administration and long-term therapy [19, 21, 22, 56, 57]. In contrast, activated platelets in concentrates release a range of active biomolecules, which influence the immunological response and promote the regeneration of affected tissues. Given that OLP is associated with degeneration of basal keratinocytes, along with subepithelial infiltration of lymphocytes and an immune-mediated course of action [12], the efficacy of PRP and i-PRF in treatment is appropriate. These agents secrete active proteins, including PDGF, TGF-β1, TGF-β2, IGF-I, EGF, fibrin, fibronectin, vitronectin, and collagen I. These proteins play important roles in cell adhesion processes, epithelial migration, and the matrix for reconstructed connective tissue [28, 31, 34, 38, 58, 59]. Nevertheless, adverse effects of platelet concentrate therapy have been documented in the literature, including localized pain or erythema at the injection site, edema, bruising, and pruritus, although these effects are transient [60]. It is essential to evaluate the strengths and weaknesses of the study before any meaningful interpretations of the results can be drawn. The analysis of the considered studies was limited by several factors, including the use of different periods between applications and the assessment of clinical presentations in disparate ways. Three studies applied the Thongprasom sign score. The study by Al-Hallak et al. preferred the REU score [49], whereas Saglam et al. used the OHIP-14 score [48]. Others utilized an individually created distinction. Furthermore, one study [47] applied PRP in the form of a gel, whereas all of the other studies injected the liquid form of the concentrates. This heterogeneity has made it challenging for the authors to analyze the findings in a unified form and present the outcomes more reliably. Conversely, all the articles included in the review employed the VAS scale to assess pain levels, which made the results of the changes considering that factor more solid and comparable. In addition to the Shalby et al. study [47], there appeared to be no randomized studies comparing the use of platelet concentrates with a placebo. The absence of a placebo control group in studies comparing different therapeutic agents increases the margin of bias in the assessment of therapeutic efficacy owing to the possibility of multiple interactions affecting the outcome of the treatment. Furthermore, in most of the participating centers, platelet levels were not measured in patients prior to the commencement of the trial. This is reflected in the number of platelet factors present in the administered agents [40, 61, 62], which has an adverse effect on the ability to investigate the therapeutic properties of equivalent levels of the relevant factors. The lack of an adequately long follow-up period in the included studies precludes an assessment of treatment stability, the intensity of agent activity throughout the therapeutic course, and a comparison with currently routinely used corticosteroids [26]. Other limitations were the small number of patients included in the trials, the limited number of randomized trials that met the inclusion criteria, and the lack of multicenter studies, which impeded the establishment of definitive, meaningful conclusions. Despite these limitations, this study also has considerable advantages, including the assessment of the risk of bias in the articles. The search was independently conducted by three authors, who used an extensive set of search criteria to minimize the risk of failing to identify significant experiments. The inclusion and exclusion criteria were applied consistently, which resulted in a limited number of articles being reviewed. It is recommended that randomized trials with larger study groups be carried out to compare various platelet concentrates both as individual treatments and as adjunctive therapies for oral lichen planus. To ensure reliable and comparable outcomes, it is essential to establish multicenter studies comprising study groups representing a significant proportion of the population with OLPs according to different ethnic, socioeconomic, and individual conditions. The use of a split-mouth design in the trial ensures the generation of reliable and comparable results, thereby reducing the risk of bias associated with individual differences. A longer follow-up period would allow monitoring of platelet concentrate activity and efficacy during subsequent disease phases. Moreover, incorporating a specific platelet level as an inclusion criterion would be beneficial, enabling the impact of a defined, consistent dosage of agents to be evaluated across a diverse patient population and reducing the potential for bias. Future studies must adhere to a standardized methodology and employ the Consolidated Standards of Reporting Trials (CONSORT) [63] approach for analysis to ensure the reliability and comparability of the results.

## Conclusions

The findings from this systematic review indicate the potential of PRP and i-PRF as innovative treatment options for oral lichen planus. These findings indicate that these therapies can markedly alleviate symptoms such as pain and discomfort while also facilitating the healing of oral tissues. Compared with conventional treatments, PRP and i-PRF represent biocompatible and less invasive alternatives, with a reduced incidence of adverse effects. However, the extant studies demonstrate a considerable degree of variability in both treatment protocols and outcomes, thereby underscoring the imperative for more rigorous and standardized clinical trials. To fully establish the efficacy and safety of these therapies, further research should focus on long-term clinical outcomes, optimal dosing strategies, and the mechanisms by which these treatments promote healing. The promising results observed to date provide a strong rationale for the continued exploration and refinement of platelet-based therapies in the management of oral lichen planus to improve patient outcomes and expand therapeutic options.

## Data Availability

Data sharing is not applicable to this article as no datasets were generated or analysed during the current study.

## Abbreviations

LP: Lichen Planus
OLP: Oral Lichen Planus
MMPs: Metalloproteinases
TNF-α: Tumor Necrosis Factor α
IL-6: Interleukin 6
COX-2: Cyclooxygenase-2
PUVA: Psoralen and ultraviolet radioation
PDT: Photodynamic therapy
PRP: Platelet-rich plasma
PDGF: Platelet-derived Growth Factors
TGF: Transforming Growth Factor
IGF: Insulin-like Growth Factor
EGF: Epidermal Growth Factor
i-PRF: Injectable Platelet-rich fibrin
RCT: Randomized Controlled Trial
PROSPERO: Prospective Register of Systematic Reviews
PRISMA: Preferred Reporting Items for Systematic Reviews and Meta-Analyses
VAS: Visual analog scale
TA: Triamcinolone acetonide
OHIP-14: Oral health impact profile scale-14
RUE: Reticulation/keratosis, erythema, and ulceration
CONSORT: Consolidated Standards of Reporting Trials
